# Juvenile Systemic Sclerosis in a Single Center: Clinical Features, Capillaroscopic Findings, Immunological Profile, and Treatment Outcomes—A Retrospective Descriptive Analysis

**DOI:** 10.3390/children13091269

**Published:** 2026-09-18

**Authors:** Maria Osminina, Vera Podzolkova, Pavel Berezhanskiy, Nadezhda Podchernyaeva, Vladimir Volnukhin, Petr Ermolinskiy, Svetlana Chebysheva, Marina Kuzina, Elena Afonina, Yulia Kostina, Viktoria Soboleva, Elizaveta Aseeva, Pavel Moldon, Vyshakie Puvaneswaran, Natalia Geppe

**Affiliations:** 1 Department of Children’s Diseases, Sechenov First Moscow State Medical University, 119435 Moscow, Russia; osminina_m_k@staff.sechenov.ru (M.O.); berezhanskiy_p_v@staff.sechenov.ru (P.B.); podchernyaeva_n_s@staff.sechenov.ru (N.P.); chebysheva_s_n@staff.sechenov.ru (S.C.); aseeva_e_v@staff.sechenov.ru (E.A.); geppe_n_a@staff.sechenov.ru (N.G.); 2 Moscow Scientific and Practical Center of Dermatovenereology and Cosmetology, 119071 Moscow, Russia; 3 Department of Physics, M.V. Lomonosov Moscow State University, 119991 Moscow, Russia; ermolinskiy.pb15@physics.msu.ru (P.E.); moldon.pavel@gmail.com (P.M.); 4 Clinic of Children’s Diseases Sechenov Center of Maternity and Childhood, 119435 Moscow, Russia; 5 N.V. Sklifosovsky Institute of Clinical Medicine, Sechenov First Moscow State Medical University, 119048 Moscow, Russia; puvanesvaran_v@student.sechenov.ru

**Keywords:** juvenile systemic sclerosis, pediatric rheumatology, nailfold capillaroscopy, interstitial lung disease, rituximab, DMARDs, treatment outcomes

## Abstract

**Highlights:**

**What are the main findings?**

- This study characterizes clinical, immunological, and microvascular features of juvenile systemic sclerosis (jSSc) in a 40-patient Russian cohort followed over 20 years, with gastrointestinal and interstitial lung involvement being the most common visceral manifestations.
- Nailfold capillaroscopy revealed giant capillaries exclusively in male patients (*p* = 0.002), and boys also showed a trend toward longer diagnostic delay, suggesting a possible association that warrants further investigation.

**What are the implications of the main findings?**

- DMARD-based regimens were associated with better outcomes in interstitial lung disease and joint involvement compared with penicillamine, although the “period effect” should be considered.
- Earlier switching to rituximab may be beneficial in refractory cases, but this requires confirmation in prospective controlled studies.

**Abstract:**

**Background/Objectives:** Juvenile systemic sclerosis (jSSc) is a rare, chronic autoimmune disease with limited pediatric data. We aimed to characterize the demographic, clinical, immunological, and microvascular features of a Russian cohort of children with jSSc, and to evaluate treatment outcomes and predictors of escalation to biologic therapy. **Methods:** A retrospective single-center study of 40 children with jSSc (36 girls, 4 boys) followed over a 20-year period (2004–2024) was conducted. Clinical assessment included the modified Rodnan Skin Score (mRSS) and the Juvenile Systemic Sclerosis Severity Score (J4S). Nailfold capillaroscopy (NFC) was performed in 12 patients. Autoantibody profiling was available for 30 patients. Two first-line regimens were compared: glucocorticosteroids with penicillamine (PA, *n* = 19) versus glucocorticosteroids with DMARDs (methotrexate, mycophenolate mofetil, or cyclophosphamide; *n* = 21). Predictors of switching to biologic therapy were identified using binary logistic regression. **Results:** Median age at onset was 9.0 years (IQR 6.0–10.0), and diagnostic delay was 12.0 months (IQR 4.0–24.0). Diffuse cutaneous jSSc predominated (82.5%). Gastrointestinal involvement was detected in 82.5%, Raynaud’s phenomenon in 87.5%, and ILD in 37.5%. NFC revealed reduced capillary density (5.26 ± 1.33/mm). Giant capillaries were observed exclusively in males (4/4 vs. 0/8, *p* = 0.002). DMARD-based regimens were associated with better outcomes than PA, particularly for joint involvement. Eleven patients (27.5%) were switched to biologic therapy, primarily rituximab. ILD and J4S > 15 were the strongest predictors of switching (probability 60–61% vs. 8–15%, *p* < 0.001). The 5-year survival was 100%; the estimated 10-year survival was 71.4%, although the latter estimate should be interpreted cautiously because of the small number of events and loss to follow-up. **Conclusions:** In our cohort, children with jSSc present predominantly with diffuse cutaneous involvement. Male patients may be at risk for more severe microvascular changes, possibly related to diagnostic delay. ILD and J4S > 15 were associated with escalation to biologic therapy, and earlier switching to rituximab may be beneficial in refractory patients. Future prospective multicenter studies are needed to validate these findings.

## 1. Introduction

Juvenile systemic sclerosis (jSSc) is a rare, chronic, multisystem autoimmune disease that manifests before the age of 16 years. It is characterized by widespread fibrosis of the skin and internal organs, obliterative microangiopathy, and immune dysregulation, with Raynaud’s phenomenon as an early and nearly universal feature [1,2]. Although the pathogenesis shares common pathways with adult systemic sclerosis (SSc), jSSc differs in several aspects, including a more aggressive disease course in some patients, a higher frequency of diffuse cutaneous involvement, and distinct patterns of organ involvement [3,4].

The rarity of jSSc poses significant challenges for clinical research. The estimated incidence is 0.05 cases per 100,000 children, and the prevalence ranges from 0.6 to 3.1 cases per 100,000 depending on the geographic region [5,6,7,8]. In the Central Federal District of the Russian Federation, jSSc accounts for less than 1% of all pediatric rheumatic diseases, although recent data from Moscow suggest a 2.2-fold increase in prevalence between 2016 and 2020, reaching 3.1 cases per 100,000 children [6]. This trend, while potentially reflecting improved awareness and diagnostic capabilities, underscores the need for continued epidemiological monitoring.

To date, the largest multicenter study of jSSc included 153 patients and was conducted by the European Paediatric Rheumatology Society [5]. Subsequent reports from individual centers have described cohorts ranging from 22 to 110 patients [9,10,11,12,13,14,15], but publications with detailed clinical, immunological, and microvascular characterization from a single pediatric rheumatology center remain scarce. This scarcity is particularly critical for understanding regional variations in disease presentation, treatment practices, and outcomes, especially in populations where diagnostic delays and access to biologic therapies may differ from Western European or North American cohorts.

Nailfold capillaroscopy (NFC) has emerged as a valuable non-invasive tool for assessing microvascular damage in SSc. In adults, specific NFC patterns (early, active, and late) correlate with disease duration, skin score, and the presence of internal organ involvement [16,17]. However, data on NFC in pediatric populations are limited. Children often exhibit less specific capillaroscopic changes, and the applicability of adult classification systems remains debated [18]. Detailed descriptions of NFC findings in jSSc, particularly their relationship to clinical features and diagnostic delay, are therefore needed [19].

The management of jSSc remains largely empiric, with treatment recommendations extrapolated from adult studies and expert consensus. Current standard care includes glucocorticoids in combination with conventional disease-modifying antirheumatic drugs (DMARDs), such as methotrexate, mycophenolate mofetil, or cyclophosphamide, depending on the predominant organ involvement [20,21]. Over the past decade, biologic agents, particularly rituximab and tocilizumab, have been increasingly used in refractory cases, especially for interstitial lung disease (ILD) and progressive skin involvement [22,23,24,25,26]. Nevertheless, the optimal timing for biologic initiation, patient selection criteria, and comparative effectiveness of different agents remain subjects of active investigation [27,28].

In this context, we conducted a retrospective single-center study of 40 children with jSSc followed at the Sechenov Center of Maternity and Childhood over a 20-year period (2004–2024). The aim of this study was to characterize the demographic, clinical, and immunological features of our cohort, to describe nailfold capillaroscopy findings and their relationship to clinical variables, and to evaluate the effectiveness of different immunosuppressive therapy regimens, with a particular focus on predictors of escalation to biologic therapy.

## 2. Materials and Methods

### 2.1. Study Design and Patients

This retrospective single-center cohort study included 40 children diagnosed with juvenile systemic sclerosis (jSSc) who were observed at the Clinic of Children’s Diseases, Sechenov Center of Maternity and Childhood, Moscow, Russia, between 2004 and 2024. All patients were diagnosed according to the preliminary classification criteria for jSSc proposed by the Pediatric Rheumatology European Society (PRES)/EULAR (European Alliance of Associations for Rheumatology) [29]. Patients with jSSc overlap syndrome were excluded from the study. The study was approved by the Local Ethics Committee of Sechenov University (protocol No. 01-25, dated 23 January 2025).

### 2.2. Clinical Assessment

All patients underwent a comprehensive clinical, physical, and instrumental evaluation at baseline and during follow-up visits. Disease activity and severity were assessed using the Juvenile Systemic Sclerosis Severity Score (J4S) and the modified Rodnan Skin Score (mRSS). The following investigations were performed routinely: complete blood count, biochemistry panel (including liver and renal function tests, creatine phosphokinase), urinalysis, electrocardiography, chest radiography, and high-resolution computed tomography (HRCT) of the chest. Echocardiography, abdominal ultrasound, and esophagogastroduodenoscopy (EGDS) were performed as part of the standard work-up. Musculoskeletal involvement was evaluated by ultrasound, radiography, or magnetic resonance imaging (MRI) of the affected joints when clinically indicated.

Gastrointestinal involvement was defined by the presence of endoscopic evidence of esophageal hypotonia, gastroesophageal reflux disease, erosive/ulcerative lesions, or esophageal stricture on EGDS. Interstitial lung disease (ILD) was defined by characteristic HRCT findings (ground-glass opacities, reticular abnormalities, or traction bronchiectasis), regardless of the presence of respiratory symptoms. Autoantibody testing was performed in 30 patients at the time of diagnosis or during the initial evaluation. The following antibodies were assessed using standard commercially available enzyme-linked immunosorbent assay (ELISA) or immunoblotting kits: antinuclear antibodies (ANA), rheumatoid factor (RF), anti-topoisomerase I antibodies (ATA/Scl-70), anti-centromere antibodies (ACA), antibodies to double-stranded DNA (anti-dsDNA), and antibodies to ribonucleoprotein 70 (anti-RNP-70), using commercial kits (Organtec, Mainz, Germany). Positivity thresholds were defined according to the manufacturers’ instructions.

### 2.3. Nailfold Capillaroscopy

Nailfold capillaroscopy (NFC) was performed in 12 patients on the volar surface of the distal phalanges of the second to fifth fingers of both hands using a video capillaroscope (Smart G-Scope G6, GenieTech, Seoul, Republic of Korea) at 250× magnification. Images were acquired and evaluated by a EULAR-certified rheumatologist. Capillaroscopy findings were assessed using standardized criteria established for systemic sclerosis and Raynaud’s phenomenon [16]. Normal capillary density was defined as ≥7 capillaries per millimeter. Capillaries with an apical diameter > 20 µm were classified as dilated, and those >50 µm as giant capillaries. Additionally, the following features were evaluated: microhemorrhages, capillary architectural disorganization (including dendritic, bushy, or tortuous shapes), perivascular edema, extravasations, and avascular zones.

### 2.4. Treatment Regimens and Definitions

**Figure 1:**
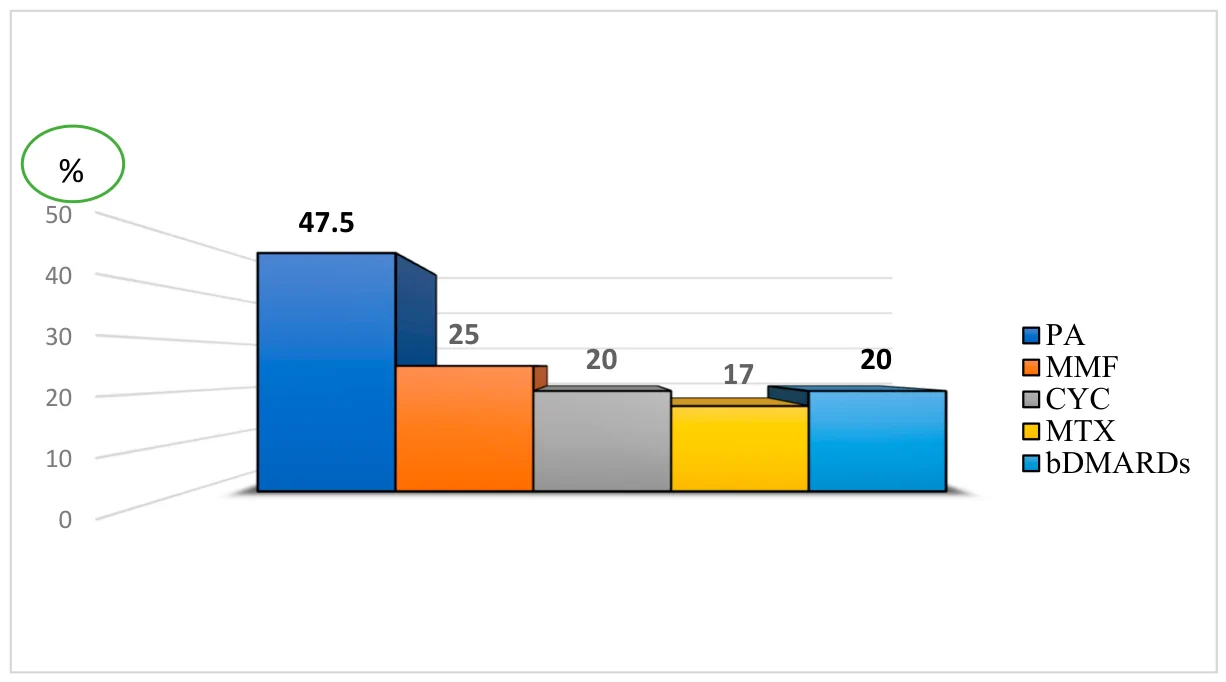
The frequency of use of the different therapeutic regimens.

All patients received combination therapy as first-line treatment, consisting of glucocorticosteroids combined with conventional disease-modifying antirheumatic drugs (DMARDs). Two initial regimens were compared. The first regimen, used predominantly in the earlier period (before 2010), comprised glucocorticosteroids in combination with penicillamine (PA). The second regimen, used predominantly after 2010, comprised glucocorticosteroids in combination with DMARDs, specifically methotrexate (MTX), mycophenolate mofetil (MMF), or intravenous pulse cyclophosphamide (CYC). The frequency of use of the different therapeutic DMARD regimens is shown in Figure 1.

Patients who did not achieve an adequate clinical response on first-line therapy were switched to a regimen containing a biologic DMARD (bDMARD), specifically rituximab (RTX) or tocilizumab (TCZ). Treatment switching was defined as the addition of a bDMARD to ongoing conventional immunosuppressive therapy. Over the past decade, the use of biologic agents has increased, particularly in patients with refractory disease.

### 2.5. Statistical Analysis

Statistical analysis was performed using Microsoft Excel and SPSS Statistics (version 26.0, IBM Corp., Armonk, NY, USA). Descriptive statistics were expressed as medians with interquartile ranges (IQR) for non-normally distributed continuous variables, and as means with standard deviations (SD) for normally distributed variables. Categorical variables were presented as frequencies and percentages.

The distribution of continuous variables was assessed for normality using the Shapiro–Wilk test. Comparisons between two groups were performed using Student’s *t*-test for normally distributed data, the Mann–Whitney U test for non-parametric data, or analysis of variance (ANOVA) for comparisons involving more than two groups. Comparisons of categorical variables were performed using the chi-square (χ^2^) test; Fisher’s exact test was applied when the expected frequency in any cell was less than 5.

Survival analysis was performed using the Kaplan–Meier method, and survival curves were compared using the log-rank test. Given the limited number of treatment-switching events, exploratory univariable logistic regression analyses were performed to assess associations between selected clinical characteristics and treatment escalation to bDMARDs. Results are presented as odds ratios (ORs) with 95% confidence intervals (CIs). No multivariable model was fitted because of the small number of events.

A two-sided *p*-value < 0.05 was considered statistically significant. Additionally, *p*-values are reported with the following notation for trend or significance: * *p* < 0.10 (trend), ** *p* < 0.05, *** *p* < 0.01, and **** *p* < 0.001.

## 3. Results

### 3.1. Demographic, Clinical and Immunological Characteristics of the Cohort

The study included 40 patients with jSSc (36 girls, 4 boys; female-to-male ratio 9:1). The median age at disease onset was 9.0 years (IQR 6.0–10.0), and the median age at first presentation to our center was 11.0 years (IQR 9.0–13.0). The median diagnostic delay, defined as the time from symptom onset to diagnosis, was 12.0 months (IQR 4.0–24.0). The diffuse cutaneous form of jSSc predominated, occurring in 33 patients (82.5%), while the limited cutaneous form was observed in 7 patients (17.5%). A summary of the demographic and clinical characteristics is presented in Table 1.

All patients (100%) presented with skin induration and joint contractures. Raynaud’s phenomenon was observed in 35 patients (87.5%), gastrointestinal involvement in 33 (82.5%), and interstitial lung disease (ILD) in 15 (37.5%). Cardiac involvement was documented in 6 patients (15.0%), and renal involvement in 1 patient (2.5%). Sicca syndrome was present in 12 patients (30.0%). The median Juvenile Systemic Sclerosis Severity Score (J4S) at baseline was 13.5 (IQR 10.0–18.0), and the mean modified Rodnan Skin Score (mRSS) was 37.4 ± 9.2 (range 11–50). New organ involvement during follow-up developed in 2 patients (5.0%).

Immunological profiling was performed in 30 patients. Antinuclear antibodies (ANA) were the most frequently detected autoantibodies, present in 23 patients (76.7%). Anti-topoisomerase I antibodies (ATA/Scl-70) were positive in 11 patients (39.3%), anti-centromere antibodies (ACA) in 3 patients (10.3%), anti-RNP-70 in 8 patients (29.6%), and rheumatoid factor (RF) in 8 patients (26.7%).

A comparative analysis of clinical and immunological features between patients with diffuse and limited cutaneous jSSc is provided in Supplementary Table S1. Briefly, patients with diffuse jSSc had significantly higher mRSS (40.0 ± 7.3 vs. 25.9 ± 1.7, *p* < 0.001), higher disease severity (J4S 13.0 vs. 18.0, *p* = 0.024), and were younger at the time of observation (10.0 ± 3.2 vs. 14.4 ± 1.1 years, *p* = 0.001). ACA positivity was observed exclusively in the limited cutaneous subgroup (42.9% vs. 0%, *p* = 0.002).

### 3.2. Nailfold Capillaroscopy Findings

Nailfold capillaroscopy (NFC) was performed in 12 patients (8 girls, 4 boys) at a median age of 13.5 years (IQR 9.75–14.25). The median diagnostic delay in this subgroup was 11.5 months (IQR 9.0–26.5), and the median disease duration before NFC was 57.5 months (IQR 28.5–96.0). At the time of NFC, all 12 patients had thickening of the skin of the fingers with contractures and periarthritis. Other clinical manifestations in this subgroup included arthralgia (83.3%), skin induration (83.3%), sclerodactyly (83.3%), hypomimia (83.3%), Raynaud’s phenomenon (75.0%), lung involvement (75.0%), and gastrointestinal involvement (75.0%). A summary of the clinical and capillaroscopic characteristics is presented in Table 2.

**Figure 2:**
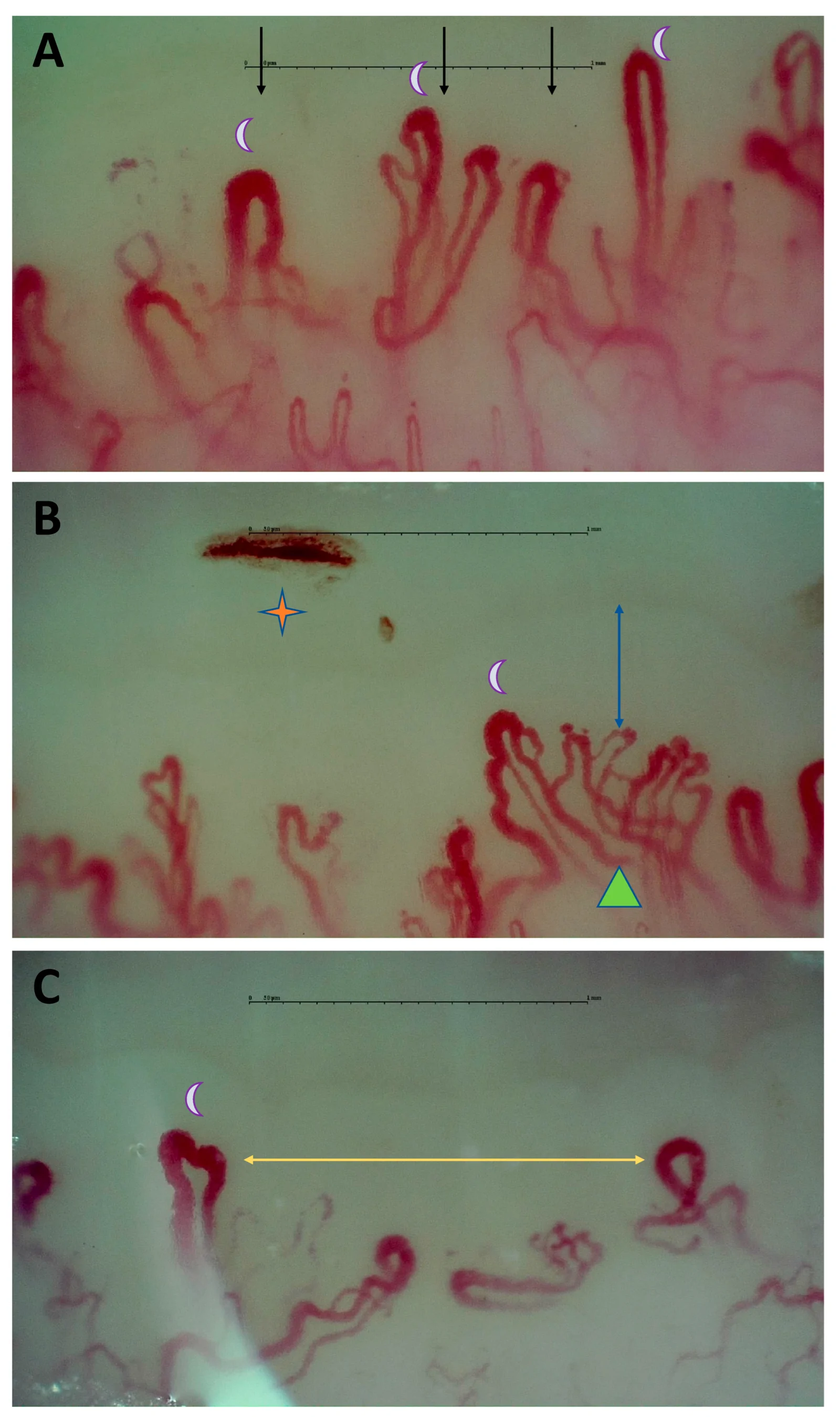
Nailfold capillaroscopy images from a 15-year-old boy with a 7-year diagnostic delay, showing marked disorganization of the capillary network: (**A**) decreased capillary density (3 capillaries/mm) with giant capillaries (crescent); (**B**) microhemorrhages (asterisk), large dendritic capillary (triangle), and perivascular edema (blue arrow); (**C**) avascular zones (yellow arrow).

The mean capillary density was significantly reduced to 5.26 ± 1.33 capillaries/mm (normal ≥7). Dilated capillaries were observed in all 12 patients (100%), while abnormal capillary morphology was observed in 11 patients (91.7%), with a bushy pattern being the most frequent morphological abnormality (11 patients, 91.7%). Microhemorrhages were present in 6 patients (50.0%), perivascular edema in 4 (33.3%), and avascular zones in a subset of patients (Figure 2).

Notably, giant capillaries were observed exclusively in male patients (4/4, 100%) and were absent in females (0/8, *p* = 0.002). Male patients also had a tendency toward lower capillary density (4.25 vs. 5.76 capillaries/mm, *p* = 0.059), more frequent perivascular edema (75.0% vs. 12.5%, *p* = 0.067), and a longer diagnostic delay (42.5 vs. 10.0 months, *p* = 0.112), although the latter did not reach statistical significance.

### 3.3. Treatment Outcomes

#### 3.3.1. First-Line Therapy: Comparison of GCS + PA Versus GCS + DMARD

All patients received combination therapy as first-line treatment. In the earlier period (before 2010), 19 patients received GCS with penicillamine (PA) (Group 1). After 2010, 21 patients received GCS with DMARSDs (MTX, MMF, or pulse CYC) (Group 2). Table 3 presents the clinical characteristics of patients receiving these two regimens. Patients in Group 2 had a significantly higher frequency of ILD on HRCT (62% vs. 11%, *p* = 0.001) and dyspnea (43% vs. 5%, *p* = 0.006), which likely reflects the more systematic use of HRCT in the later period rather than a true difference in disease severity. However, patients in Group 2 also had a higher disease severity score (J4S 16.0 vs. 11.2, *p* = 0.002)—Table 3.

**Figure 3:**
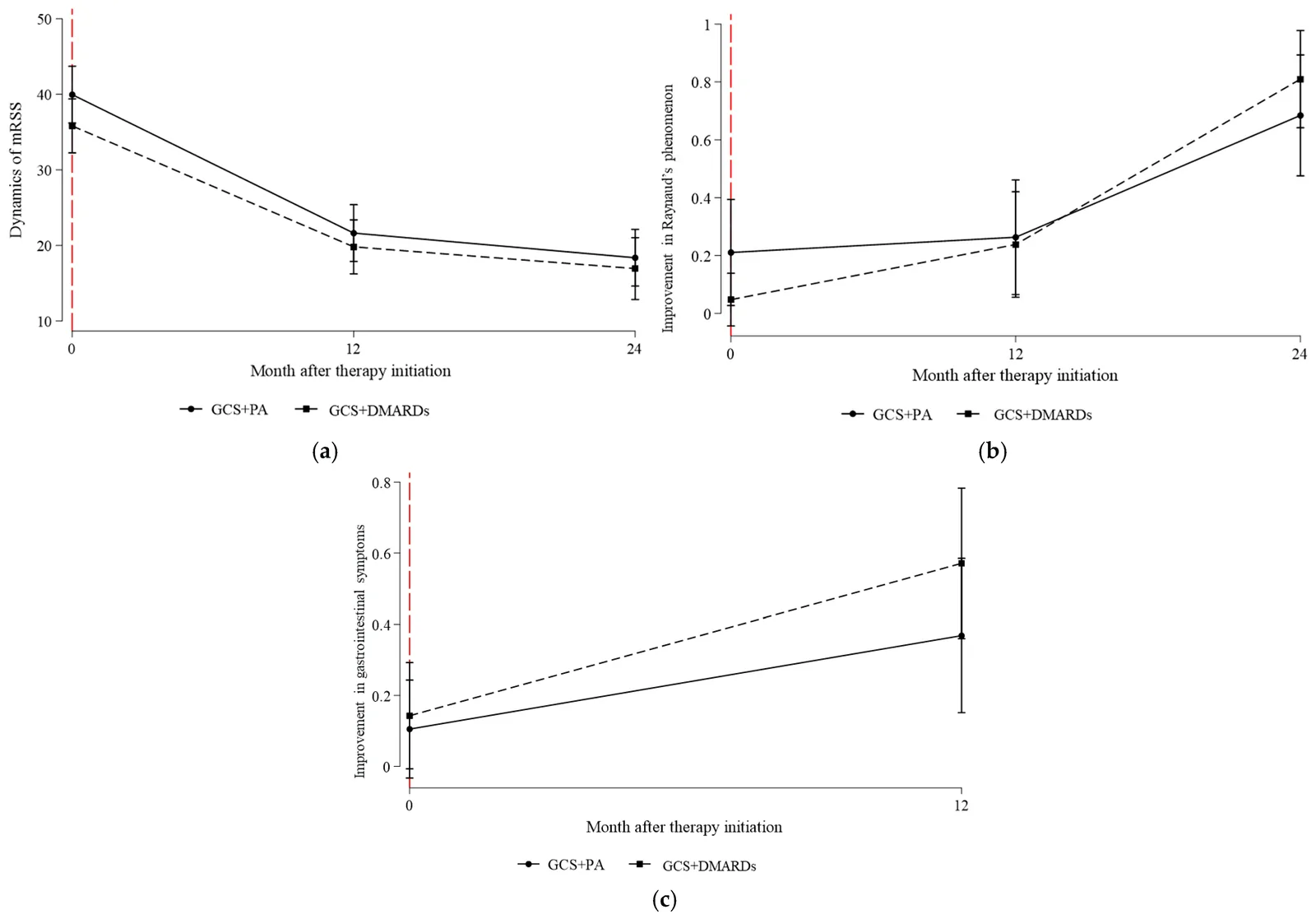
Effect of the two first-line therapy regimens on: (**a**) modified Rodnan Skin Score (mRSS) over 24 months; (**b**) Raynaud’s phenomenon over 24 months; (**c**) GI symptoms over 12 months. Abbreviations: GCS—glucocorticosteroids, DMARDs—disease-modifying antirheumatic drugs, PA—penicillamine.

**Figure 4:**
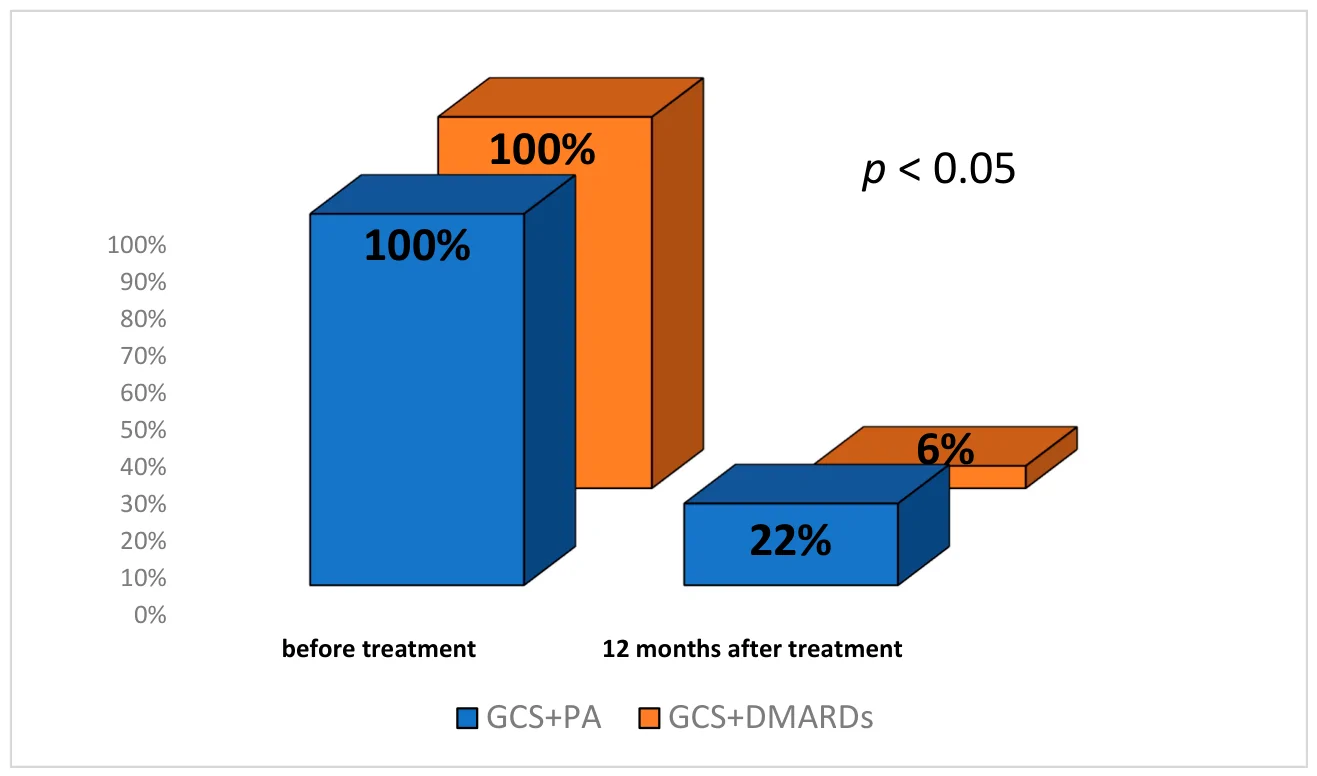
Frequency of joint contractures before and after 12 months of treatment with the two first-line therapy regimens.

**Figure 5:**
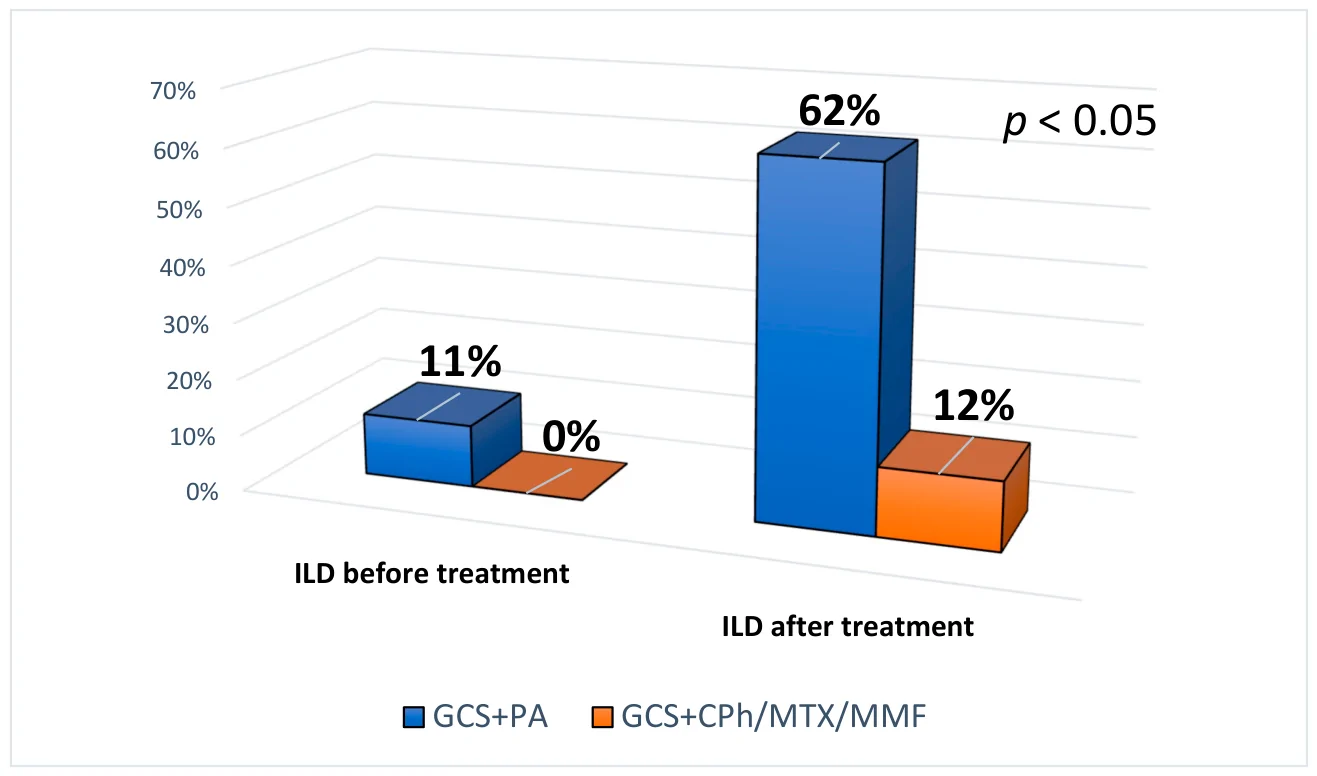
Frequency of ILD on HRCT before and after 12 months of treatment with the two first-line therapy regimens.

Both regimens resulted in clinical improvement (Figure 3). The GCS + DMARD regimen showed a trend toward greater efficacy in reducing skin fibrosis and joint involvement, although these differences did not reach statistical significance. Notably, the cytostatic regimen associated with a more pronounced reduction in ILD (as assessed by HRCT) and joint contractures compared with PA (Figure 4 and Figure 5).

#### 3.3.2. Predictors and Outcomes of Switching to Biologic Therapy

Eleven patients (27.5%) were switched to a biologic-containing regimen (GCS + MMF + rituximab in 10 patients; GCS + tocilizumab in 1 patient). All switches were performed in patients who had initially received the GCS + DMARDs regimen. The median time from initiation of first-line therapy to switching was 12.0 months (range 6–72, mean 25.4 ± 24.1 months). The median time from disease onset to switching was significantly longer: 26.0 months (range 8–72, mean 36.2 ± 26.3 months).

The main indication for switching was progression of ILD or lack of clinical improvement in pulmonary function during initial therapy (in 10 patients switched to rituximab). One patient was switched to tocilizumab due to progressive cutaneous and articular disease.

Clinical characteristics of patients who switched to biologic therapy versus those who did not are shown in Table 4. Switchers had a significantly higher rate of ILD (82% vs. 21%, *p* < 0.001), dyspnea (55% vs. 14%, *p* = 0.007), HRCT-confirmed ILD (82% vs. 21%, *p* < 0.001), and genetic thrombophilia (36% vs. 7%, *p* = 0.020). The disease severity index (J4S) was also significantly higher in the switching group (17.1 vs. 12.5, *p* = 0.011).

In exploratory univariable logistic regression analyses, HRCT-confirmed ILD was associated with higher odds of switching to biologic therapy (OR 17.25, 95% CI 2.92–101.90; *p* = 0.002). A J4S score > 15 points was also associated with higher odds of treatment switching (OR 12.80, 95% CI 2.48–65.97; *p* = 0.002) (Table 5). Given the small number of switching events, these findings should be interpreted as exploratory associations rather than independent predictors. These data indicate that lung involvement and greater disease severity are the principal factors driving escalation to biologic therapy.

**Figure 6:**
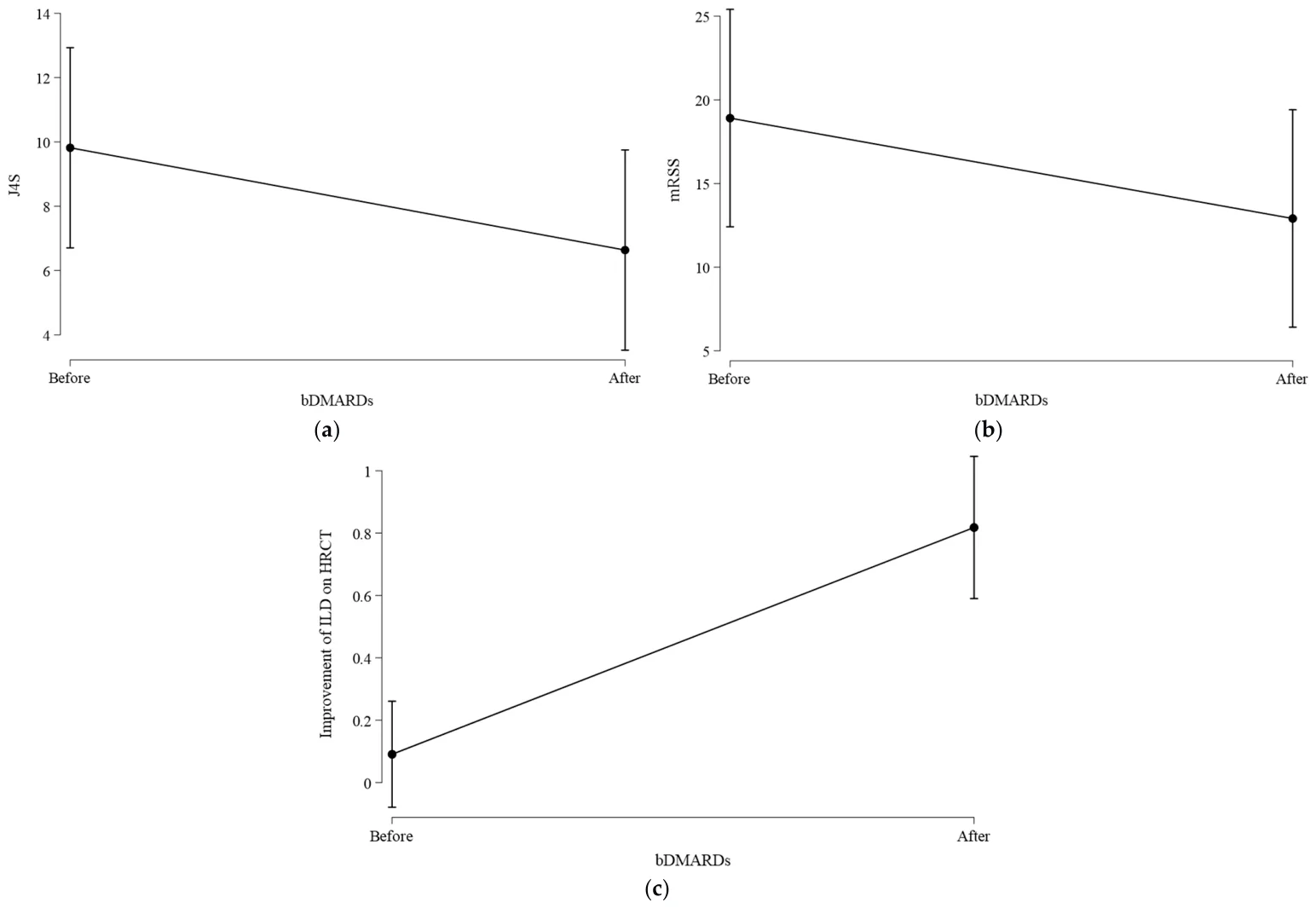
Changes in (**a**) J4S, (**b**) mRSS, and (**c**) HRCT-confirmed ILD before and after switching to bDMARD therapy.

All 11 patients who switched to a biologic-containing regimen demonstrated clinical improvement, primarily manifested by stabilization or regression of ILD on HRCT, a reduction in mRSS and a decrease in the J4S (Figure 6).

#### 3.3.3. Survival Analysis

The duration of follow-up ranged from 24 to 130 months (mean 55 ± 21 months). Three patients (7.5%) died during the observation period. Fatal outcomes occurred at 74 months (2 patients) and 125 months (1 patient) after disease onset. The immediate causes of death were multiple organ failure secondary to ILD (*n* = 2) and pancarditis with nephropathy (*n* = 1). All deaths were in female patients with the diffuse cutaneous form of jSSc.

**Figure 7:**
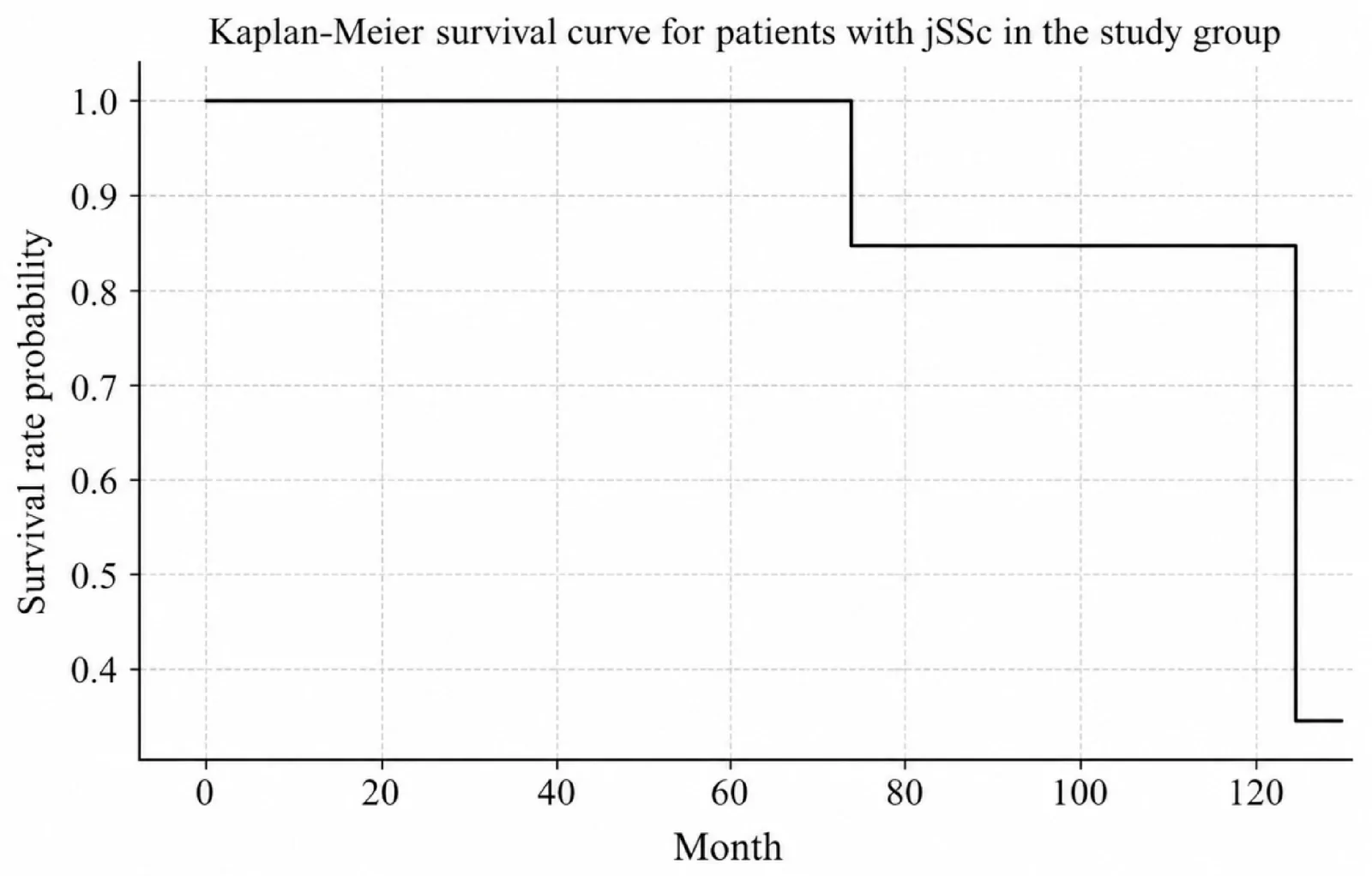
Kaplan–Meier survival curve for patients with jSSc in our cohort.

The 5-year survival rate was 100%. The estimated 10-year survival rate was 71.4%; however, this estimate is limited by the small sample size and loss to follow-up (25% of patients dropped out after 6 years, as the observation period was limited to childhood, i.e., up to 18 years of age). The Kaplan–Meier survival curve is shown in Figure 7.

## 4. Discussion

In this retrospective single-center study of 40 children with juvenile systemic sclerosis (jSSc), we characterized the demographic, clinical, immunological, and microvascular features, evaluated the effectiveness of different immunosuppressive therapy regimens and identified predictors of escalation to biologic therapy of a Russian cohort followed over a 20-year period. Our findings confirm that jSSc in our population predominantly affects girls, presents most frequently with the diffuse cutaneous form, and shares many clinical and immunological features with previously reported cohorts. However, several observations merit further discussion, including the high frequency of gastrointestinal involvement, possible differences in the diagnostic delay and the capillaroscopic differences between sexes, the diagnostic biases influencing treatment comparisons, and the predictors of response to biologic therapy.

The demographic and immunological characteristics of our cohort are broadly consistent with those reported in previous studies, although some differences merit comment (Table 6). The proportion of diffuse cutaneous jSSc in our study (82.5%) was substantially higher than in most other reports, with the notable exception of the Martini multicenter cohort (90.8%) [5]. The exclusion of patients with overlap syndrome from both studies likely explains this similarity, as overlap cases tend to have a higher proportion of limited cutaneous involvement and distinct autoantibody patterns. By contrast, studies that included overlap syndrome patients reported diffuse jSSc in only 30–54% of cases [6,9,12,13].

**Figure 8:**
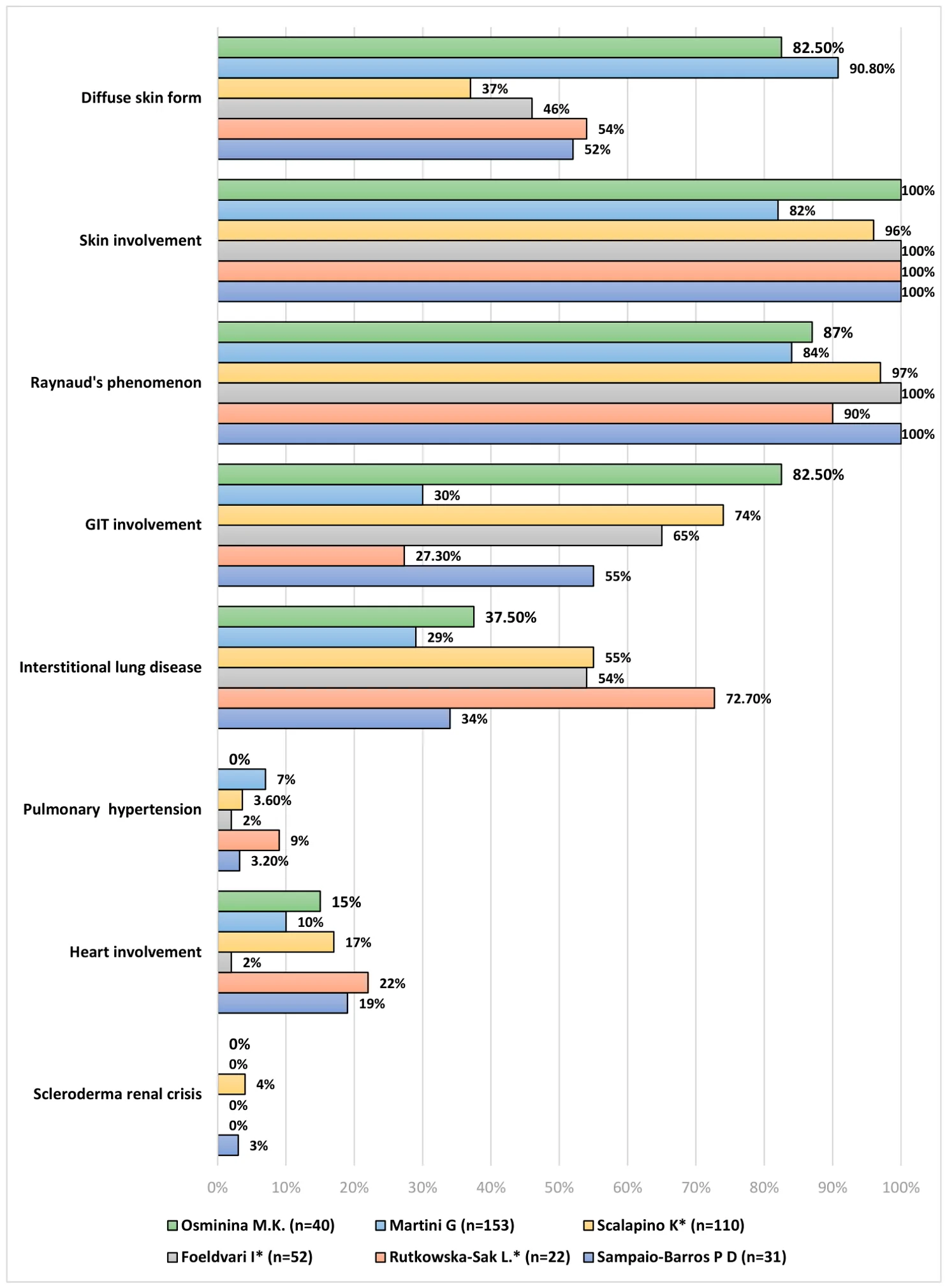
Comparison of major clinical symptoms of jSSc between the present study and previously published cohorts [5,6,9,11,12]. Studies marked with an asterisk (*) included patients with overlap syndrome.

The diagnostic delay in our cohort (median 12 months, IQR 4–24 months) aligns with the 18 months reported by Martini [5] and is shorter than the >2 years reported in 20% of patients in the study by Herrick et al. [1]. The frequency of gastrointestinal involvement in our cohort (82.5%, Figure 8) was notably higher than in most previous reports (30–74%) [9,10,11,12,13,14,15]. We attribute this difference primarily to our diagnostic approach: esophagogastroduodenoscopy (EGDS) was part of the mandatory examination protocol for all patients at baseline and was repeated during follow-up when clinically indicated. By contrast, Rutkowska-Sak et al. [12] reported that EGDS was performed only in patients with longstanding disease or gastrointestinal symptoms, likely resulting in underestimation of gastrointestinal involvement. Similarly, other studies have reported only the prevalence of gastroesophageal reflux disease (GERD) rather than comprehensive endoscopic findings [13,14]. Our experience suggests that routine endoscopic evaluation identifies a substantially higher burden of gastrointestinal disease in jSSc, including esophageal hypotonia, erosive/ulcerative lesions, and strictures.

Interstitial lung disease (ILD) was detected in 37.5% of our patients, which is comparable to the 29–37% reported in studies that excluded overlap syndrome [5,11]. Studies that included patients with overlap syndrome reported higher ILD frequencies (55–70%) [6,9,12,13], highlighting the importance of strict inclusion criteria when comparing cohorts. Pulmonary arterial hypertension (PAH) was not detected in any of our patients, consistent with its low reported prevalence in pediatric jSSc (up to 7%) [30]—Figure 8.

The autoantibody profile in our cohort closely resembles that of the multicenter study by Martini et al. [5], with ANA being the most frequently detected antibody (76.7% vs. 81%) and ATA positivity (39.3% vs. 34%) more common than ACA positivity (10.3% vs. 7.1%) (Table 6). This pattern differs from adult systemic sclerosis, in which ATA and ACA are detected more frequently [1,2]. We observed a statistically significant association between ACA positivity and the limited cutaneous subtype (42.9% vs. 0% in diffuse, *p* = 0.002), consistent with the well-established association in adults. However, unlike the study by Foeldvari et al. [20], we did not find significant differences in ATA positivity between diffuse and limited subgroups, possibly due to the small number of patients with the limited form in our cohort (*n* = 7).

A detailed comparison of clinical and immunological features between diffuse and limited jSSc in our study versus those reported by Foeldvari et al. [31] is provided in Supplementary Table S1. Both studies showed a predominance of girls and diffuse jSSc. However, patients in our cohort had substantially higher mRSS (40 vs. 18 for diffuse; 25.9 vs. 9 for limited), suggesting more severe skin involvement. Joint contractures and gastrointestinal involvement were significantly more frequent in both diffuse and limited subgroups in our study compared with Foeldvari’s cohort (*p* < 0.05). The autoantibody profiles differed significantly between the two studies, likely because Foeldvari’s study included patients with overlap syndrome (14% of the cohort). In contrast to Foeldvari’s findings, we did not observe significant differences in autoantibody positivity between jSSc subtypes in our cohort.

In our study, nailfold capillaroscopy (NFC) confirmed significant microvascular damage in our patients, with a mean capillary density of 5.26 ± 1.33 capillaries/mm, well below the normal threshold of 7/mm. Dilated capillaries and abnormal bushy morphology were the most frequent findings, reflecting neovascularization in response to progressive capillary loss. Interestingly, we observed that giant capillaries were present exclusively in male patients, all of whom demonstrated this severe microvascular abnormality, whereas none of the female patients did (*p* = 0.002). Additionally, the delay in diagnosis was greater in boys: 42.50 [27.50; 60.50] months versus 10.0 [8.25; 12.25] months in girls, although these differences didn’t reach the threshold of statistical significance, *p* = 0.112. These observations may reflect a lower clinical suspicion of jSSc in boys, resulting in delayed diagnosis, initiation of therapy and consequently, more severe microvascular damage. However, it should be noted that these findings are based on a relatively small subgroup of 12 patients who underwent NFC, which limits the generalizability of our conclusions. Therefore, these results should be interpreted with caution and require confirmation in larger pediatric cohorts.

The applicability of the adult NFC classification systems (early, active, and late patterns) proposed by Cutolo et al. [32] to pediatric populations has been questioned. In our cohort, most patients exhibited non-specific or mixed patterns, which aligns with previous observations that children with jSSc often do not fit neatly into the adult classification framework [18]. This may reflect the ongoing development of the microcirculation in children or differences in the tempo of microvascular injury [19]. Given the limited data on NFC in jSSc, we believe that quantitative parameters may be more informative than pattern-based classification in pediatric patients. Our findings suggest that NFC is a valuable tool for assessing microvascular damage in jSSc, particularly in patients with diagnostic delay.

All patients in the study received combination therapy either with GCS and PA or GCS in combination with DMARDs (MTX/CYC/MMF). Switching between immunosuprressive agents was common during follow-up. Increasing use of immunosuppressive agents and declining use of PA was likewise reported by previous studies [33,34].

A comparative analysis of the two initial first-line therapy regimens revealed a reduction in disease activity with both regimens. Although no statistically significant differences were observed overall, the GCS + DMARD regimen was significantly more effective than GCS + PA in terms of ILD and joint involvement. However, it is important to acknowledge that this comparison is heavily confounded by a “period effect”: the GCS + PA regimen was predominantly used in the pre-2010 era, whereas GCS + DMARDs was introduced after 2010. During this later period, the routine utilization of high-resolution computed tomography (HRCT) and other modern diagnostic tools became standard practice, enabling earlier detection and more precise monitoring of internal organ involvement. Therefore, the superior outcomes observed in the post-2010 group should be interpreted not as a direct pharmacological head-to-head comparison, but rather as a reflection of the overall evolution of comprehensive rheumatological care, which includes advances in imaging, monitoring protocols, and supportive therapies. Our findings should therefore be framed as demonstrating how the modernization of pediatric rheumatology practice has led to improved clinical trajectories in jSSc, rather than as evidence of the superiority of a specific drug regimen.

Treatment-resistant cases were managed in accordance with current SHARE recommendations [35,36,37]. A treatment switch to a biologic-containing regimen was performed in 27.5% of patients (*n* = 11). All switches occurred in the group that initially received the GCS + DMARD regimen. Treatment switching was performed in patients with ILD, a J4S > 15 points, or genetic thrombophilia. Although lung involvement is a component of the J4S composite index, not all clinicians routinely use this scale. Therefore, we chose to emphasize HRCT findings, which are more universally available and directly influence treatment decisions and prognosis in clinical practice. However, we acknowledge that J4S > 15 and ILD on HRCT are not entirely independent, as the J4S composite index inherently incorporates cardiopulmonary assessments. Accordingly, these findings should be interpreted as exploratory clinical associations rather than independent predictors, and the wide confidence intervals reflect the limited sample size. In all 11 patients, switching to the GCS + MMF + rituximab regimen resulted in favorable clinical outcomes, primarily through stabilization or regression of ILD on chest CT and a reduction in the J4S. The median time from disease onset to switching was 26.0 months, and the mean duration of biologic therapy was 36 months. Our real-world data are consistent with and validate the SHARE recommendations supporting the use of rituximab in refractory jSSc [22,26,36,37]. These findings suggest that earlier switching to biologic therapy may become standard practice when initial therapy is ineffective. However, the optimal timing for treatment switching remains uncertain, given the relatively recent introduction of biologic DMARDs in systemic sclerosis and the rarity of jSSc.

The 5-year survival rate in our cohort was 100%, which is comparable to the 98% reported in the European registry [20]. However, the estimated 10-year survival rate was 71.4%, which is lower than the 87.4% reported in the European registry. Several factors may explain this discrepancy. The 10-year survival estimate was based on a small number of events and was additionally affected by loss to follow-up, which limits its precision and reliability. First, our cohort had a high proportion of patients with the diffuse cutaneous form (82.5%), which is associated with more severe disease and higher mortality. Second, the diagnostic delay in our cohort, particularly in male patients, may have contributed to more advanced disease at presentation. Third, the smaller sample size and loss to follow-up (25% after 6 years, as observation was limited to childhood) limit the reliability of the 10-year estimate.

**Figure 9:**
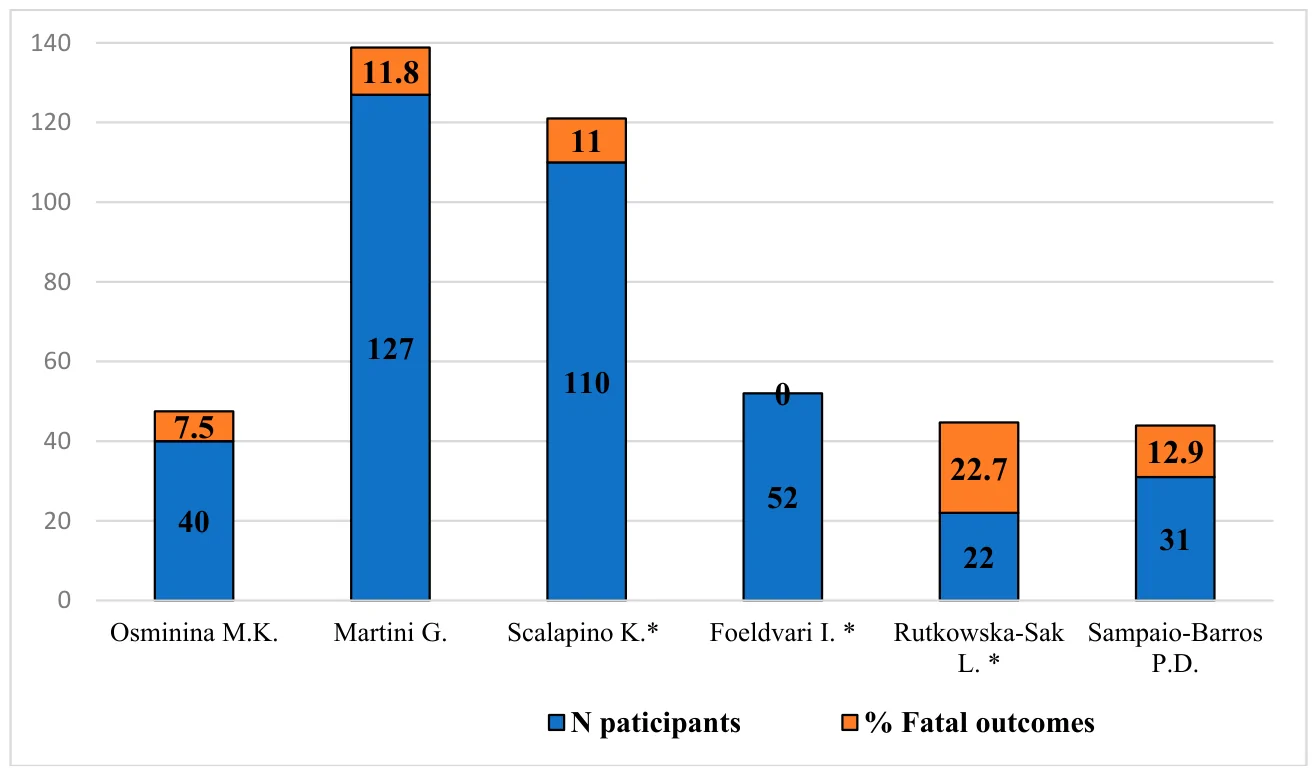
Mortality rates in patients with jSSc reported across different cohorts. Studies marked with an asterisk (*) included patients with overlap syndrome [5,6,9,11,12].

The overall mortality rate in our cohort was 7.5% (3/40 patients). This is comparable to the 8–12.9% reported in other studies [5,10,11] (Figure 9), with the exception of Rutkowska-Sak et al. [12], who reported a mortality rate of 22.7%, likely due to prolonged follow-up extending into adulthood, when mortality from SSc is higher [1,2].

All deaths in our study occurred in female patients with the diffuse form, due to multiple organ failure secondary to ILD, pancarditis, and nephropathy. These findings underscore the severity of diffuse jSSc and the need for aggressive monitoring and treatment of internal organ involvement.

## 5. Limitations

Several limitations of this study should be acknowledged. First, the retrospective design and the single-center nature of the study limit the generalizability of our findings and introduce potential selection and information biases. Second, the small sample size, particularly in the subgroups (male patients, limited cutaneous form, and the NFC subgroup), limits the statistical power of some analyses and the reliability of subgroup comparisons. Third, the long study period (2004–2024) introduces temporal biases, including changes in diagnostic criteria, imaging techniques (particularly HRCT), treatment protocols, and referral patterns. As discussed above, the apparent superiority of the cytostatic regimen in ILD is likely confounded by the more frequent use of HRCT in the later period. Fourth, the immunological profile was not available for all patients, and the NFC examination was performed in only 12 patients, limiting the generalizability of these findings. In addition, anti-SS-A/Ro antibodies were not included in the autoantibody panel; therefore, we cannot exclude the possibility of associated Sjögren’s disease in patients with sicca symptoms, although this was interpreted as a clinical manifestation of jSSc rather than an independent condition. Anti-RNA polymerase III antibodies were not assessed due to technical and financial limitations; future studies should include them for a more complete autoantibody profile. Fifth, the loss to follow-up (25% after 6 years) limits the reliability of the long-term survival estimates.

Despite these limitations, our study provides valuable real-world data on the clinical features, microvascular changes, and treatment outcomes of jSSc in a Russian population, and highlights the importance of diagnostic vigilance, particularly in male patients, and the potential benefits of earlier escalation to biologic therapy in refractory cases.

## 6. Conclusions

This 20-year single-center study of 40 children with jSSc confirms that the clinical and demographic features of Russian patients are largely comparable to those reported in other cohorts. Male patients may be at risk for more severe microvascular changes, possibly related to diagnostic delay. DMARD-based regimens appeared more effective than penicillamine-based regimens, although the apparent advantage in ILD is confounded by diagnostic bias. ILD and J4S > 15 were associated with escalation to biologic therapy. Our observations suggest that earlier switching to rituximab may be beneficial in refractory patients, but this cannot be established from the current uncontrolled retrospective data. While the 5-year survival was favorable, the 10-year survival may be lower than in European cohorts, highlighting the need for improved long-term follow-up. Future prospective multicenter studies are needed to validate our findings and define optimal treatment strategies.

## Figures and Tables

**Table 1:** Demographic and clinical characteristics of patients with jSSc (*n* = 40).

Parameter	Value
Number of patients	40
Gender, female/male (ratio)	36/4 (9:1)
Age at disease onset, years, Me (IQR)	9.0 (6.0–10.0)
Age at observation, years, Me (IQR)	11.0 (9.0–13.0)
Diagnostic delay, months, Me (IQR)	12.0 (4.0–24.0)
Disease subtype, *n* (%):	
Diffuse cutaneous form	33 (82.5)
Limited cutaneous form	7 (17.5)
Skin induration, *n* (%)	40 (100)
Joint contractures, *n* (%)	40 (100)
Raynaud’s phenomenon, *n* (%)	35 (87.5)
Gastrointestinal involvement, *n* (%)	33 (82.5)
Interstitial lung disease, *n* (%)	15 (37.5)
Pulmonary arterial hypertension, *n* (%)	0
Cardiac involvement, *n* (%)	6 (15.0)
Renal involvement, *n* (%)	1 (2.5)
Sicca syndrome, *n* (%)	12 (30.0)
J4S score, Me (IQR)	13.5 (10.0–18.0)
mRSS, mean ± SD (range)	37.4 ± 9.2 (11–50)
New organ involvement during follow-up, *n* (%)	2 (5.0)
Immunological profile (*n* = 30)	
Antinuclear antibodies (ANA)	23 (76.7)
Anti-topoisomerase I (ATA/Scl-70)	11 (39.3)
Anti-centromere antibodies (ACAs)	3 (10.3)
Anti-RNP-70	8 (29.6)
Rheumatoid factor (RF)	8 (26.7)
Anti-dsDNA	2 (6.7)

Abbreviations: jSSc, juvenile systemic sclerosis; Me, median; IQR, interquartile range; SD, standard deviation; J4S, Juvenile Systemic Sclerosis Severity Score; mRSS, modified Rodnan Skin Score.

**Table 2:** Clinical and capillaroscopic characteristics of patients who underwent nailfold capillaroscopy (*n* = 12).

Parameter	Total (*n* = 12)	Girls (*n* = 8)	Boys (*n* = 4)	*p* Value
Demographics				
Age at NFC, years, Me (IQR)	13.5 (9.75–14.25)	13.5 (9.75–14.50)	13.5 (12.0–14.25)	0.931
Diagnostic delay, months, Me (IQR)	11.5 (9.0–26.5)	10.0 (8.25–12.25)	42.5 (27.75–60.50)	0.112
Disease duration before NFC, months, Me (IQR)	57.5 (28.5–96.0)	45.0 (28.5–76.0)	84.0 (58.0–96.0)	0.610
Capillaroscopy findings				
Capillary density, per mm, mean ± SD	5.26 ± 1.33	5.76 ± 1.30	4.25 ± 0.96	0.059
Dilated capillaries, *n* (%)	12 (100)	8 (100)	4 (100)	–
Abnormal morphology, *n* (%)	11 (91.7)	7 (87.5)	4 (100)	1.000
Bushy pattern, *n* (%)	11 (91.7)	7 (87.5)	4 (100)	1.000
Dilated apical pattern, *n* (%)	3 (25.0)	3 (37.5)	0	0.491
Spire-like pattern, *n* (%)	3 (25.0)	2 (25.0)	1 (25.0)	1.000
Crossing capillaries, *n* (%)	2 (16.7)	2 (25.0)	0	0.515
Giant capillaries, *n* (%)	4 (33.3)	0	4 (100)	**0.002**
Microhemorrhages, *n* (%)	6 (50.0)	3 (37.5)	3 (75.0)	0.545
Perivascular edema, *n* (%)	4 (33.3)	1 (12.5)	3 (75.0)	0.067
Perivascular extravasates, *n* (%)	2 (16.7)	1 (12.5)	1 (25.0)	1.000
Laboratory parameters				
Leukocytes, ×10^9^/L, mean ± SD	7.42 ± 2.77	6.36 ± 2.56	9.54 ± 1.96	0.055

Abbreviations: NFC, nailfold capillaroscopy; Me, median; IQR, interquartile range; SD, standard deviation. Statistically significant *p*-values are shown in bold.

**Table 3:** Comparison of clinical characteristics between patients receiving GCS + PA and GCS + DMARDs as first-line therapy.

Parameter	Group 1: GCS + PA (*n* = 19)	Group 2: GCS+ DMARDs (*n* = 21)	*p* Value
Skin induration, %	100	100	NA
Skin fibrosis, %	58	86	0.049
Muscle damage, %	68	76	0.582
Raynaud’s phenomenon, %	79	95	0.119
Digital necrosis, %	26	67	**0.011**
Joint contractures, %	100	100	NA
Gastrointestinal involvement, %	74	94	0.105
ILD (HRCT findings), %	11	62	**0.001**
–Dyspnea, %	5	43	**0.006**
Cardiac involvement, %	16	18	0.881
mRSS, mean ± SD	39.9 ± 6.8	35.8 ± 9.2	0.118
J4S, mean ± SD	11.2 ± 4.8	16.0 ± 4.6	**0.002**
Fatal outcome, %	11	5	0.489

Abbreviations: GCS, glucocorticosteroids; PA, penicillamine; DMARD, disease-modifying antirheumatic drug; ILD, interstitial lung disease; HRCT, high-resolution computed tomography; mRSS, modified Rodnan Skin Score; J4S, Juvenile Systemic Sclerosis Severity Score; NA, not applicable. Statistically significant *p*-values are shown in bold.

**Table 4:** Comparison of clinical characteristics between patients with and without treatment switching to biologic therapy.

Parameter	No Switching (*n* = 29)	Switching to bDMARD (*n* = 11)	*p* Value
ILD (HRCT findings), %	21	82	**<0.001**
Dyspnea, %	14	55	**0.007**
Genetic thrombophilia, %	7	36	**0.020**
J4S, mean ± SD	12.5 ± 5.2	17.1 ± 3.8	**0.011**
Digital necrosis, %	38	73	**0.049**
mRSS, mean ± SD	38.2 ± 8.1	36.7 ± 9.3	0.631
ATA positivity, %	38	64	0.145

Abbreviations: bDMARD, biologic disease-modifying antirheumatic drug; ILD, interstitial lung disease; HRCT, high-resolution computed tomography; J4S, Juvenile Systemic Sclerosis Severity Score; mRSS, modified Rodnan Skin Score; ATA, anti-topoisomerase I antibodies. Statistically significant *p*-values are shown in bold.

**Table 5:** Exploratory univariable associations with switching to biologic therapy.

Clinical Characteristic	OR	95% CI	*p* Value
HRCT-confirmed ILD	17.25	2.92–101.90	0.002
J4S > 15 points	12.80	2.48–65.97	0.002

Abbreviations: ILD, interstitial lung disease; HRCT, high-resolution computed tomography; J4S, Juvenile Systemic Sclerosis Severity Score.

**Table 6:** Clinical forms, sex distribution and age at disease onset of jSSc according to different authors.

Study	Girls (%)	Diffuse jSSc (%)	Mean Age at Onset, Years	ANA	ATA	ACA	RNP-70	RF
Own data (*n* = 30)	90	82.5	9	76.7	39.3	10.3	29.6	26.7
Martini G (*n* = 153) [5]	72.5	90.8	8.1	81	34	7.1	***	17
Scalapino K * (*n* = llO) [7]	83	37	10	97	20	8	18	***
Foeldvari I * (*n* = 52) [8]	75	46	14	23	33	2	15	***
Rutkowska-Sak L * (*n* = 22) [11]	68	54	6.8	86.7	18.2	31.8	9	22
Sampaio-Barros PD (*n* = 31) [10]	90	52	12.7	***	22	16	***	***
Jeong Ji E * (*n* = 31) [12]	61	30	10	100	53.8	-	18.2	-

* patients with overlap syndrome were included. *** parameter not studied. - parameter not detected.

## Data Availability

The data presented in this study are available on request from the corresponding author due to being part of an ongoing study.

## Supplementary Materials

The following supporting information can be downloaded at: https://www.mdpi.com/article/10.3390/children13091269/s1, Table S1: Comparison of the clinical and immunological characteristics of patients with diffuse jSSc and limited jSSc between our study and the study by I. Foeldvari.
