# Supplementary material for: Juvenile Systemic Sclerosis in a Single Center: Clinical Features, Capillaroscopic Findings, Immunological Profile, and Treatment Outcomes—A Retrospective Descriptive Analysis
Source: Children (Basel). 2026 Sep 18;13(9):1269. doi: 10.3390/children13091269 (PMC13605266; doi:10.3390/children13091269)
Supplement: Supplementary file 1 [file children-13-01269-s001.zip › children-4535625-supplementary.pdf]

Table S1. Comparison of the clinical and immunological characteristics of patients with diffuse jSSc and limited jSSc between our study and the study by I. Foeldvari.

	**Diffuse jSSC**			**Limited jSSc**		
**Parameter**	**Osminina,** ***n*** **= 33**	**Foeldvari,** ***n*** **= 57**	***p*-Value**	**Osminina,** ***n*** **= 7**	**Foeldvari,** ***n*** **= 22**	***p*-Value**
Female patients	90%	66%	0.0082	42%	14%	0.0649
Diffuse type of SSc	82%	72%	0.2939	-	-	-
Modified Rodnan Skin Score (mean)	40	18	-	25.9	9	-
Raynaud's phenomenon	84%	91%	0.3562	100%	86%	0.3704
Digital ulcers	39%	60%	0.0536	71%	23%	*0.0174*
Joint contractures	100%	42%	<0.0001	100%	55%	*0.0209*
Gastrointestinal involvement	78.9%	38%	0.0002	85.7%	18%	*0.0012*
ILD	36%	41%	0.6326	28%	22%	0.7181
PAH	0%	7%	0.1585	0%	5%	0.5684
Cardiac involvement	12%	3%	0.1577	28%	23%	0.7827
Scleroderma renal crisis	0%	0%	-	0%	0%	-
ANA positivity	76%	24%	<0.0001	72%	4%	*<0.0001*
ATA positivity	39.4%	29%	0.3650	71.4%	31%	0.0920
ACA positivity	6.1%	3.4%	0.6221	0%	9%	1.000

Note: Statistically significant differences are shown in italics.
